# Tubular Cellulose Composite as a Vehicle for the Development of Meat Products with Low Nitrite Content

**DOI:** 10.17113/ftb.62.01.24.8154

**Published:** 2024-03

**Authors:** Athanasia Panitsa, Theano Petsi, Maria Kanellaki, Athanasios A. Koutinas, Panagiotis Kandylis

**Affiliations:** 1 Food Biotechnology Group, Department of Chemistry, University of Patras, Panepistimioupoli, Rio Achaias, 26504 Patras, Greece; 2 Department of Food Science and Technology, Ionian University, Vergoti Avenue, Argostoli, 28100 Kefallonia, Greece

**Keywords:** leaf celery, potassium nitrite, veal, tubular cellulose composite, microbiological analysis, low nitrite content

## Abstract

**Research background:**

Nitrite salts are among the most used preservatives in meat products as they ensure their safe consumption. Despite their positive effects on food safety and stability, many side effects on human health have been reported, leading to the need to reduce their use. Therefore, the aim of this study is to produce veal products with low nitrite content through low diffusion of potassium nitrite and to study their microbiological characteristics.

**Experimental approach:**

Edible tubular cellulose from leaf celery was produced and KNO_2_ was encapsulated in this material. This was achieved in two ways: by impregnation of tubular cellulose in a KNO_2_ solution under stirring and using starch gel as a stabilizer. Two samples of impregnated cellulose were applied on the surface of two veal samples of which one was stored at room temperature and the other at 3 °C. Similarly, two samples of cellulose with starch gel were applied on the surface of two veal samples of which one was stored at room temperature and the other at 3 °C. The KNO_2_ diffusion in different depths of the meat was measured and its effect on the microbiological characteristics of the meat was evaluated. Τhe experiment was carried out in duplicate.

**Results and conclusions:**

A satisfactory percentage of about 70 % of the initially encapsulated amount of KNO_2_ was diffused in the meat, while the rest remained in the pores of the delignified leaf celery. The migrating amount of KNO_2_ proved to be effective in preserving meat, as the microbiological load decreased significantly (especially within the first 12 h, from a decrease of 0.6 log CFU/g up to 2.4 log CFU/g).

**Novelty and scientific contribution:**

The demand for meat products with low nitrite content is constantly increasing and the results of the present study are promising for the development of this technology in scale-up systems and on an industrial scale. This innovative approach could lead to products with controlled diffusion of the preservatives.

## INTRODUCTION

Nowadays, there is a growing consumer demand for environmentally friendly food production with fewer chemical additives and higher nutritional value. This trend also applies to the meat industry, where the reduction or even complete elimination of chemical preservatives is of great importance. Nitrite and nitrate have traditionally been used in meat products mainly for their effectiveness against *Clostridium botulinum*, both cells and spores, and to a lesser extent against other bacteria (*1*, *2*). However, the beneficial effects of nitrite and nitrate in meat products are not limited to their antimicrobial activity. They also contribute to the development and maintenance of the characteristic red colour, to the development of characteristics flavours, to the prevention of oxidation, and generally to the improvement of the organoleptic and sensory properties of meat products, in particular of dried meat products (*2*, *3*). In addition, all these properties of nitrite and nitrate have no effect on the muscle enzymes such as aminopeptidases and lipases and their contribution to the quality development of meat. Finally, nitrate can also be used by some microorganisms to produce nitrite through their nitrate reductase activity (*2*).

Despite all these positive contributions of nitrite and nitrate in the meat industry, a link between nitrite and N-nitrosamines (highly carcinogenic compounds) has been established since the early 1970s (*4*, *5*), leading to an increasing number of studies looking for possible nitrite substitutes (*6*, *7*). Plant extracts have been suggested as an ideal alternative, as many of them are natural sources of nitrate and nitrite (*8*), thus providing a ’clean label’ for the meat product without actually eliminating nitrate and nitrite. However, there are also approaches to reduce nitrite and nitrate content by adding natural antimicrobials such as spices, plant and fruit extracts, lactate, bacteriocins or even bioprotective and colour-enhancing starter cultures (*9*, *10*), while the effects of the antioxidant properties of plant extracts and their application on the shelf life and quality of meat products have also been investigated (*11*). However, despite the great potential of these alternatives, to date there is no single additive available on the market that can fulfil all the above-mentioned functions of nitrite, which means that nitrite remains unique to the meat industry (*7*).

Recently, tubular cellulose from edible materials and food by-products has been evaluated as an efficient carrier of chemical preservatives and their delivery in food products (*12*, *13*). In the case of meat products, the development of a new process for the delivery of nitrite in meat products based on a composite with edible plant tubular cellulose has been proposed (*12*). In that study (*12*), the encapsulation material was characterized and evaluated with regard to the efficiency of encapsulation of the preservative and its diffusion into pork. The results of these studies (*12*, *13*) were promising and satisfactory, as a controlled release of each preservative in the used food products was achieved with good efficacy in inhibiting microbiological growth. The cellulosic raw materials used for encapsulation are edible and widely used in cooking. However, they showed promising porosity properties after delignification, which was an important characteristic for successful encapsulation. For this reason, the aim of the present work is to investigate the gradual release of potassium nitrite in another food matrix in the way we had used in our previous study (*12*). As it seems to be an effective method for preserving pork, our aim is to investigate its effect on veal, which has several different properties compared to pork. In this way, we can better evaluate how the food affects the release of potassium nitrite and, most importantly, its preservation using our proposed method. Although the use of nitrite is very common in research manuscripts and in industry, the development of methods to reduce its content by targeted diffusion is very rare.

## MATERIALS AND METHODS

### Materials and chemicals

Leaf celery and veal were bought from local markets of Patras (Greece), ΚNΟ_2_ was supplied from PENTA (Nové Město, Czech Republic), NaOH pellets were supplied from Lachner (Neratovice, Czech Republic), NitriVer 2 nitrite reagent powder pillows from Hach (Manchester, UK) and food grade corn starch powder was supplied from Wintersun Chemical (Ontario, CA, USA).

### Substrate preparation: edible tubular cellulose from leaf celery

Leaf celery was used as a raw material for the production of edible tubular cellulose. Before delignification, leaf celery was dried in the air (also known as hang drying), as it was not pulverized. The edible tubular cellulose was prepared after the dried leaf celery (1 kg) was treated with 3 L NaOH solution (10 g/L) with heating (70 °C for 3 h) and then filtered (sieve with *d*(pore)=0.25 cm), washed with hot deionized water, freeze-dried (Labtech Daihan LFD-S; Namyangju, South Korea) and pulverized (*12*). This process, called delignification, leads to the formation of pores on the surface of the substrate that ensure the encapsulation of the chemical preservative.

### Potassium nitrite encapsulated in edible tubular cellulose

In order to entrap KNO_2_, 1.5 g of edible tubular cellulose were added to 40 mL of nitrite solution (6.3 g/L) under continuous stirring for 2 h. The samples were filtered (sieve with *d*(pore)=0.25 cm), then freeze-dried and kept at room temperature for further use.

To produce the nitrite encapsulated in edible tubular cellulose covered with starch gel, 0.45 g of corn starch was added to 9 mL of deionized water and heated up to 90 °C. Before stabilization at 40 °C, 2 mL of potassium nitrite solution (75 g/L) were added. The mixture was then added dropwise to 1.5 g of edible tubular cellulose and freeze-dried.

### Potassium nitrite diffusion in meat

**Fig. S1:**
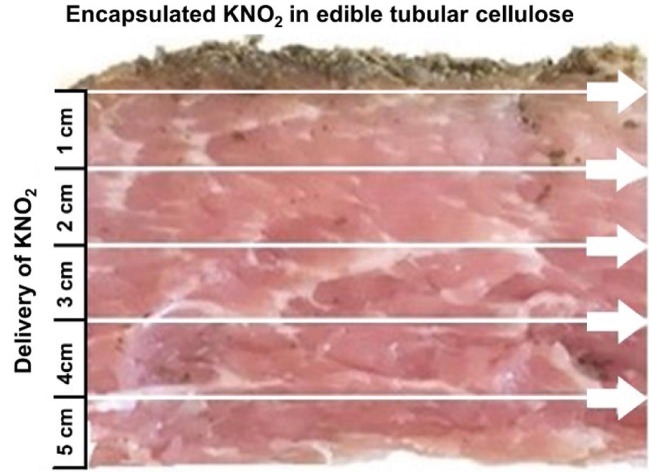
Illustration of meat sampling for the controlled release of KNO_2_ study

A nearly rectangular piece (about *l*=16 cm, *b*=8 cm, *m*=600 g) of *biceps femoris* muscle from the veal leg (subcutaneous fat was removed) was used. Two samples of nitrite encapsulated in edible tubular cellulose–starch gel composite and two samples of nitrite encapsulated in edible tubular cellulose were evenly distributed over a flat and wide surface of each meat sample to obtain nitrite mass fraction of 150 mg/kg. The meat was then wrapped in a PVC food film suitable for temperatures from 20 to -30 °C and for contact with all foods according to EU specifications (*14*). A meat sample treated with nitrite encapsulated in edible tubular cellulose and another treated with nitrite encapsulated in edible tubular cellulose–starch gel composite were stored in a cold chamber (Incucell incubator; MMM Medcenter Einrichtungen GmbH, München, Germany) at 3 and 25 °C. A control meat sample without nitrite treatment was also stored at both temperatures. Meat samples were stored at 3 °C for up to 240 h or at 25 °C for up to 48 h. These two temperatures were chosen because they are the most common temperatures at which meat is either stored to preserve it for a certain time or at which various meat products are matured. Samples cut in 5 cm wide and 9 cm long slices were taken with a scalpel from each meat sample at the two temperatures (meat treated with nitrite encapsulated in edible tubular cellulose, meat treated with nitrite encapsulated in edible tubular cellulose–starch gel composite, and control meat sample) at the same time. To investigate the diffusion of KNO_2_ at different depths of the meat, each slice was cut into 5 equal parts. Each part was 1, 2, 3, 4 or 5 cm away from the upper part of the meat, where the two nitrite samples were spread (Fig. S1). The experiment was done in duplicate.

### Nitrite determination

Nitrite content was determined spectrophotometrically at 585 nm using commercially available reagent powder pillows (NitriVer 2 nitrite reagent powder pillows) and a Hach DR/2400 spectrophotometer (Baltimore, MD, USA) according to the iron(II) sulfate method (*12*, *15*). Specifically, 5 g of each sample (taken as described above) were slurried and then 200 mL of deionised water were added and the entire mixture was heated to 100 °C for 10 min. The mixture was then homogenized while maintaining the temperature constant. The homogenate was diluted with 100 mL of water and filtered by straining in a tulip followed by vacuum filtration to obtain the meat extracts. The extracts were analyzed spectrophotometrically at 585 nm without further dilution to determine the amount of KNO_2_ that was diffused in the meat (*12*).

### Microbiological analysis

Meat samples (treated with nitrite encapsulated in cellulose–starch gel composite and control) of 10 g (from the top of the sample to 5 cm in depth) were used for the microbiological analysis. After homogenization in a stomacher bag mixer (BagMixer 400; Interscience, Cantal, France) and serial dilution in sterile Ringer’s solution (Merck, Darmstadt, Germany), the samples were used for the enumeration of the following microbial groups: mesophilic bacteria (plate count agar (*16*)), yeasts and moulds (potato dextrose agar (*16*)), enterobacteriaceae (Violet Red Bile Glucose Agar (*17*)), coliforms (Violet Red Bile Agar (*16*)), lactic acid bacteria (MRS agar (*18*)) and lactococcus (M-17 agar (*19*)).

### Statistical analysis

All experiments and analyses were carried out in duplicate. Significance was established at p<0.05. The statistical significance of the results was analyzed with ANOVA, and Tukey’s honestly significant difference (HSD) test was used to determine significant differences between the results. Coefficients, ANOVA tables and significance (p<0.05) were computed using Statistica v. 12 (*20*).

## RESULTS AND DISCUSSION

The safety of meat products is a very important issue and therefore numerous research studies are conducted to improve the safety of such products (*21*, *22*). In addition, the overuse of synthetic/chemical antimicrobial agents like potassium nitrate/nitrite has raised some concerns in the scientific community but also among consumers (*2*). For example, many studies focus on plant extracts as substitutes for nitrites that could effectively preserve meat products and that have no side effects on human health. However, many factors need to be considered when using such substances. First of all, the extraction solvents must be toxicologically safe and fulfil the specific purity criteria. Permitted extraction solvents include ethanol, acetone, butyl acetate and, under certain conditions, hexane and dichloromethane can also be used. Ethanol is the most common solvent as it is nontoxic and has GRAS status, so it is safe to use in food. If methanol or other cheaper solvents than ethanol are used, their residues must be removed from the extract. Apart from the extraction solvent and technique, the raw material is also important. The efficiency of the extract depends on its origin. Specifically, it depends on the growth phase of the plant used and also on the part of the plant used in the extraction. The cost of this method must also be considered. The solvent, plant drying technique, evaporation of large amounts of solvents and total energy consumption are factors that can significantly increase the cost of this method. We must also take into account the fact that many plant extracts contain nitrite and therefore the addition of such extracts could have the same side effects on the human body as the consumption of nitrites from chemical sources. Finally, the ineffectiveness of the extracts in preventing the development of certain types of microorganisms has been observed in some cases (*23*, *24*). Other methods to reduce or partially replace nitrite in meat include treatment with gamma rays, X-rays or electron beams. Among these, gamma irradiation seems to be the most effective method. However, many obstacles limit its use such as the distinctive smell that can be produced, the fact that it is not suitable for raw meat and its products and that it can lead to nutrient loss. Moreover, it may be a commercially unacceptable processing method for the consumer community as it is harmful and requires special precaution. Plasma-treated water is another method that can be used as a substitute for nitrite or nitrate. It is a modern method that aims to reduce processing time and protect the environment. However, it is suitable for sterilization of unpacked meat and meat products and results in damage to protein structure (*25*, *26*). Other methods such as cooking and active packaging can be used in limited cases but have many disadvantages, such as the fact that long-term steaming during cooking leads to an increased nitrite content in the cured meat, has high energy requirements and the packaging materials pollute the environment (*25*). Following the trend to reduce nitrite in meat and meat products, our research group has proposed in a previous study the use of edible nanotubular cellulose as a carrier for the diffusion of potassium nitrite in meat, but also of sodium benzoate in juice (*12*, *13*). Since each food matrix is different and differences also occur among meat samples from different sources, the present study investigated the effect of the addition of potassium nitrite on veal and in particular on its microbiological properties.

### Potassium nitrite diffusion in meat

Table 1 shows the effect of time on the diffusion of potassium nitrite at 3 °C at different depths in the veal. More specifically, nitrite encapsulated in edible tubular cellulose or edible tubular cellulose–starch gel composite was evenly distributed over a flat and wide surface of the meat, which was then stored at 3 °C. The results show that the storage time significantly affected the distribution of nitrite in the meat. Nitrite diffusion increased both in concentration and in depth of the veal. In addition, the use of starch gel in the material led to a reduction of nitrite diffusion of up to 19 % after 2 and 5 days and 10 % after 10 days of storage at 3 °C. Indeed, statistical analysis proved that support (edible tubular cellulose with or without starch gel) significantly (p<0.05) affected nitrite diffusion during all days of storage, while a significant effect of the support with depth was also observed. Similar results were observed in pork (*12*). Therefore, the use of starch gel could offer the possibility for controlled nitrite diffusion.

**Table 1:** Effect of storage (at 3 °C) on the diffusion (%) of nitrite in different depths of veal samples treated with nitrite encapsulated in edible tubular cellulose or edible tubular cellulose–starch gel composite

Depth/cm	*t*(storage)/day							Effect of time
0.5	1	1.5	2	3	5	10
Diffusion(nitrite)/%						
In edible tubular cellulose						
1	0^a^	(12.4±2.9)^b,B^	(16.4±2.1)^bc,C^	(18.3±1.7)^bcd,C^	(21.8±2.4)^cd,C^	(25.8±1.4)^de,D^	(31.8±2.4)^e,C^	***
2	0^a^	(7.2±1.5)^a,B^	(13.4±2.1)^bc,BC^	(11.0±1.7)^b,B^	(12.5±1.2)^bc,B^	(18.2±1.4)^cd,C^	(21.8±2.4)^d,B^	***
3	0^a^	(0)^a,A^	(8.2±1.1)^b,B^	(9.1±0.9)^bc,B^	(9.2±1.2)^bc,B^	(12.4±1.4)^cd,B^	(15.9±1.2)^d,B^	***
4	0^a^	(0)^a,A^	(0)^a,A^	(3.0±0.9)^b,A^	(3.3±0.1)^b,A^	(11.5±0.1)^d,B^	(5.9±1.2)^c,A^	***
5	0^a^	(0)^a,A^	(0)^a,A^	(0)^a,A^	(0)^a,A^	(3.8±0.1)^a,A^	(3.3±0.1)^a,A^	ns
SUM	0^a^	(19.6±1.5)^b^	(37.9±1.1)^c^	(41.6±3.4)^cd^	(46.9±2.4)^d^	(71.7±1.4)^e^	(78.6±2.4)^e^	***
Effect of sample depth		**	***	***	***	***	***	
In edible tubular cellulose–starch gel composite								
1	0^a^	(4.7±0.6)^ab,B^	(9.6±1.5)^bc,C^	(14.5±2.3)^cd,C^	(16.1±1.5)^cd,D^	(20.9±2.3)^de,C^	(25.1±2.7)^e,C^	***
2	0^a^	(4.3±0.1)^ab,B^	(8.6±0.1)^bc,BC^	(12.9±0.1)^cd,C^	(11.8±1.5)^c,CD^	(11.3±2.3)^c,AB^	(16.2±1.1)^d,B^	***
3	0^a^	(0)^a,A^	(5.4±1.5)^b,B^	(6.4±0.1)^b,B^	(7.5±1.5)^b,BC^	(12.9±0.1)^c,B^	(13.5±2.7)^c,AB^	***
4	0^a^	(0)^a,A^	(0)^a,A^	(0)^a,A^	(4.3±0.1)^b,AB^	(6.8±0.5)^c,A^	(8.1±0.5)^d,A^	***
5	0^a^	(0)^a,A^	(0)^a,A^	(0)^a,A^	(0)^a,A^	(6.4±0.1)^b,A^	(7.7±0.1)^b,A^	ns
SUM	0^a^	(9.0±0.6)^b^	(23.6±3.0)^b^	(33.8±2.3)^c^	(39.6±4.5)^c^	(58.2±0.5)^d^	(70.6±1.6)^e^	***
Effect of sample depth		***	***	***	***	***	***	
Effect of diffusion system		**	***	**	*	***	ns	
Effect of diffusion system and sample depth		**	**	*	*	**	**	

Values are shown as mean±standard deviation (*N*=2). Mean values within a row with different lowercase letters in superscript differ significantly (p<0.05). Mean values (SUM is excluded) within a column with different uppercase letters in superscript differ significantly (p<0.05); *p<0.05, **p<0.01, ***p<0.001, ns=not significant

**Fig. 1:**
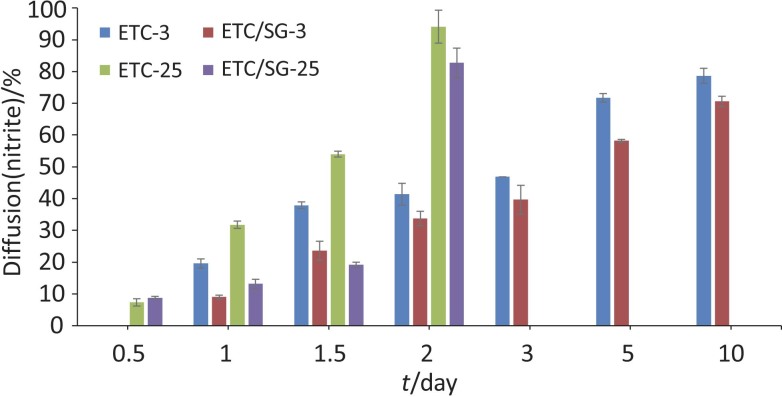
Effect of storage time and temperature (3 and 25 °C) on the diffusion of nitrite in different depths of veal samples treated with potassium nitrite encapsulated in edible tubular cellulose (ETC-nitrite) and potassium nitrite encapsulated in edible tubular cellulose-starch gel composite (ETC/SG-nitrite). ETC-3 and ETC-25=meat sample treated with potassium nitrite encapsulated in edible tubular cellulose stored at 3 and 25 °C respectively, ETC/SG-3 and ETC/SG-25=meat sample treated with potassium nitrite encapsulated in edible tubular cellulose-starch gel composite stored at 3 and 25 °C respectively

The diffusion of potassium nitrite in meat also took place at room temperature. Table 1 shows the results at 3 °C, while Fig. 1 compares the results at room temperature and at 3 °C. Thus, similar results were observed for the experiments performed at room temperature (25 °C). More specifically, starch gel led to delayed diffusion of nitrite compared to edible tubular cellulose. However, in all cases a faster deeper diffusion of nitrite was observed due to the high temperatures (Fig. 1). Diffusion is the most important mass transfer mechanism during salting/curing of a meat sample (either with NaCl or/and with nitrites). The effective diffusivity can be calculated using diffusion models. When modelling, this parameter can be considered constant or dependent on specific process or product conditions. It is therefore significantly affected by the operating temperatures. An increase in temperature increases the thermal energy of the molecules, which leads to an increase in the diffusion rate of the molecules. Thus, the dependence of diffusivity on temperature is generally described by the Arrhenius equation (*27*). The viscoelastic properties of the solid matrix also change strongly with temperature, and a softening of the structure is observed in accordance with the increase in temperature (*28*).

### Microbiological characteristics of the veal

The most important issue with meat products is their microbiological safety. For this reason, in the present study, the microbiological load of the veal treated with nitrite encapsulated in edible tubular cellulose–starch gel composite was evaluated during storage at 3 °C for up to 10 days (Table 2). These meat samples had a high microbiological load to observe the effect of nitrite diffusion. In all microbial groups, the use of nitrite encapsulated in edible tubular cellulose–starch gel composite and storage time had a significant (p<0.001) effect on their numbers compared to the control sample. In addition, apart from lactic acid bacteria, a combined effect of these two factors was also observed.

**Table 2:** Microbiological analysis of control meat sample and sample treated with nitrite encapsulated in edible tubular cellulose–starch gel composite for a period of 10 days at 3 °C

*t*/h	*N*/(log CFU/g)						
Sample	Mesophilic bacteria	Yeast Molds	Εnterobacteriaceae	Coliforms	LAB	Lactococci
0	Control	(5.3±0.7)^bc^	(4.4±0.2)^abc^	(3.7±0.1)^def^	(3.9±0.2)^cd^	(3.5±0.4)^a^	(5.8±0.3)^cd^
12	Control	(5.47±0.07)^c^	(4.7±0.2)^abcd^	(3.8±0.3)^def^	(4.0±0.1)^cd^	(3.7±0.3)^a^	(6.0±0.5)^cde^
	ETC/SG-nitrite	(3.99±0.03)^a^	(3.9±0.2)^a^	(2.0±0.1)^a^	(1.55±0.06)^a^	(2.97±0.04)^a^	(3.5±0.3)^a^
24	Control	(5.5±0.1)^c^	(5.55±0.08)^def^	(3.74±0.04)^def^	(4.0±0.2)^cd^	(4.1±0.2)^a^	(6.2±0.3)^c^
	ETC/SG-nitrite	(4.36±0.04)^ab^	(4.01±0.08)^ab^	(2.1±0.1)^a^	(2.0±0.1)^a^	(3.3±0.2)^a^	(3.9±0.2)^a^
36	Control	(5.9±0.1)^cd^	(6.0±0.4)^fg^	(3.7±0.2)^def^	(4.0±0.2)^cd^	(5.83±0.06)^bc^	(6.2±0.2)^c^
	ETC/SG-nitrite	(5.0±0.1)^abc^	(5.0±0.2)^bcde^	(2.4±0.2)^ab^	(2.0±0.1)^a^	(4.1±0.2)^a^	(4.1±0.1)^ab^
48	Control	(6.00±0.07)^cd^	(6.9±0.1)^gh^	(3.7±0.3)^def^	(4.0±0.1)^cd^	(6.7±0.3)^bcd^	(7.0±0.6)^def^
	ETC/SG-nitrite	(5.5±0.2)^bc^	(5.79±0.06)^ef^	(2.9±0.1)^bc^	(3.0±0.2)^b^	(5.5±0.3)^b^	(5.4±0.6)^bc^
72	Control	(6.0±0.3)^cd^	(7.0±0.5)^h^	(3.9±0.3)^ef^	(4.3±0.2)^d^	(7.0±0.2)^cde^	(7.03±0.07)^def^
	ETC/SG-nitrite	(5.5±0.1)^bc^	(5.96±0.08)^fg^	(3.04±0.08)^bcd^	(3.1±0.2)^b^	(5.8±0.2)^bc^	(6.0±0.6)^cde^
120	Control	(6.07±0.06)^cd^	(7.1±0.2)^h^	(4.1±0.2)^f^	(4.6±0.1)^de^	(7.9±0.4)^de^	(7.2±0.2)^e^
	ETC/SG-nitrite	(5.8±0.3)^c^	(5.9±0.5)^efg^	(3.2±0.3)^cde^	(3.3±0.4)^bc^	(6.8±0.6)^cde^	(6.76±0.08)^cdef^
240	Control	(8.2±0.5)^e^	(7.1±0.2)^h^	(5.1±0.2)^g^	(5.1±0.2)^e^	(8.0±0.3)^e^	(8.0±0.1)^f^
	ETC/SG-nitrite	(7.1±0.2)^de^	(5.1±0.1)^c^	(3.4±0.2)^cdef^	(3.4±0.3)^bc^	(7.0±0.4)^cde^	(6.9±0.3)^def^
Effect of KNO_2_		***	***	***	***	***	***
Effect of time		***	***	***	***	***	***
Effect of KNO_2_/Time		*	**	***	***	ns	***

Values are shown as mean±standard deviation (*N*=2). ETC/SG-nitrite=meat sample treated with potassium nitrite encapsulated in edible tubular cellulose from leaf celery and starch gel composite; Control=meat sample without potassium nitrite addition; LAB=lactic acid bacteria; ^a-h^Mean values within a column with different letters in superscript differ significantly (p<0.05); *p<0.05, **p<0.01, ***p<0.001, ns=not significant

Comparing the control sample with the sample treated with nitrite encapsulated in cellulose–starch gel composite after 10 days of storage, the number of mesophilic bacteria decreased from 8.2 to 7.1 log CFU/g, of yeast and moulds from 7.1 to 5.1 log CFU/g, of enterobacteriacae from 5.1 to 3.4 log CFU/g, coliforms from 5.1 to 3.4 log CFU/g, LAB from 8.0 to 7.0 log CFU/g and lactococci from 8.0 to 6.9 log CFU/g. A remarkable inhibition of microbial growth was thus achieved after 10 days. The results also show that the microbiological content of the control sample increased continuously during storage. In the sample treated with nitrite encapsulated in cellulose–starch gel composite, a significant decrease of all microorganisms was observed in the first 12 h, ranging from a decrease of 0.6 log CFU/g (yeasts, moulds and LAB) to 2.4 log CFU/g (coliforms). However, after that a continuous slight increase in all counts was observed up to 48–72 h. Interestingly, such growth of microorganisms was not detected in the samples of pork treated with nitrite encapsulated in cellulose–starch gel composite (*12*), probably due to the higher initial load in the present study and different diffusion constant of nitrite in pork and beef samples (*29*). In a recent study with dry fermented sausages, the absence of nitrifying salts and their replacement with pork liver autohydrolysate allowed the growth of spoilage microorganisms (*30*). In another study with Cantonese sausage, the reduction in nitrite content led to an increase in the total viable counts and Gram-positive cocci, a decrease in the valuable lactic acid bacteria, and also changed the aroma characteristics. The results showed that it is very difficult to determine the appropriate amount of nitrites in such products (*31*).

In the present study, it would be very interesting to determine the effect of the sample size of meat on these results. Table 1 shows that the first diffusion of nitrite occurs at a depth of 4 cm after day 3 and at 5 cm after day 5, which probably affects the microbiological analysis of the samples. More specifically, a sample with a depth of 4 cm is expected to have a better microbiology than the 5 cm sample of the present study. Therefore, a meat sample of up to 8 cm (with nitrite encapsulated in edible tubular cellulose–starch gel composite added on both upper and bottom surfaces) can be better preserved as it contains a sufficient amount of the preservative that effectively inhibits the growth of microorganisms.

The results of our previous and this study show that this type of preservation by encapsulating potassium nitrite in an edible substrate so that it is gradually released in the meat can prevent further growth or reduce the microbiological load, while a significant part of the preservative remains encapsulated in the cellulose substrate, thus reducing the amount that is eventually consumed. This is due to the fact that cellulose is a raw material that is not digested by the human body. Among the complex biochemical processes that occur in the human body, cellulose is eventually excreted from the digestive system without having been metabolized, especially when it is in the form of micro- and nanocrystalline cellulose, as is the case after delignification (*32*). Also, many studies have shown that the use of micro- and nanocellulose in food is safe without toxic side effects on human health. Microcellulose fibre and crystals are already used in processed foods as fillers, enhancers, for example, and have been classified as safe (GRAS) for human consumption by the US Food and Drug Administration (FDA) (*33*).

## CONCLUSIONS

As nitrite content in meat and its products poses a major risk to human health, many studies have focused on reducing nitrite content by various methods. However, the disadvantages of these methods limit their application. Therefore, our team has proposed an alternative method to reduce nitrite content. The results of the present study on veal confirm the results on pork regarding the efficiency of edible tubular cellulose or cellulose–starch gel composite for the diffusion of nitrite in meat samples. Regarding the microbiological stability, different behaviour was observed in each meat sample, which should be taken into account in the scaling-up. The diffusion of nitrite is satisfactory at a meat depth of up to 4 cm. Therefore, a meat sample of up to 8 cm (treated with nitrite encapsulated in edible tubular cellulose–starch gel composite on all surfaces) can be preserved as it contains a sufficient amount of the preservative to effectively inhibit the growth of microorganisms. The edible tubular cellulose–starch gel can be removed before the consumption/processing of the meat, or even consumed. The proposed method is an effective way of food preservation without toxic or any undesirable effect on human health. The results are promising, but further research is needed to evaluate the effects of this method on the colour and antioxidant properties of meat, and to develop the technology for KNO_2_ diffusion in meat and meat products in scale-up systems.
